# Indirect Measurement of Ground Reaction Forces and Moments by Means of Wearable Inertial Sensors: A Systematic Review

**DOI:** 10.3390/s18082564

**Published:** 2018-08-05

**Authors:** Andrea Ancillao, Salvatore Tedesco, John Barton, Brendan O’Flynn

**Affiliations:** Tyndall National Institute, University College Cork, Lee Maltings Complex, Dyke Parade, T12R5CP Cork, Ireland; salvatore.tedesco@tyndall.ie (S.T.); john.barton@tyndall.ie (J.B.); brendan.oflynn@tyndall.ie (B.O.)

**Keywords:** biomechanical modelling, ground reaction forces, inertial measurements, inertial measurement units (IMU), kinetics, machine learning, wearable sensors

## Abstract

In the last few years, estimating ground reaction forces by means of wearable sensors has come to be a challenging research topic paving the way to kinetic analysis and sport performance testing outside of labs. One possible approach involves estimating the ground reaction forces from kinematic data obtained by inertial measurement units (IMUs) worn by the subject. As estimating kinetic quantities from kinematic data is not an easy task, several models and protocols have been developed over the years. Non-wearable sensors, such as optoelectronic systems along with force platforms, remain the most accurate systems to record motion. In this review, we identified, selected and categorized the methodologies for estimating the ground reaction forces from IMUs as proposed across the years. Scopus, Google Scholar, IEEE Xplore, and PubMed databases were interrogated on the topic of Ground Reaction Forces estimation based on kinematic data obtained by IMUs. The identified papers were classified according to the methodology proposed: (i) methods based on direct modelling; (ii) methods based on machine learning. The methods based on direct modelling were further classified according to the task studied (walking, running, jumping, etc.). Finally, we comparatively examined the methods in order to identify the most reliable approaches for the implementation of a ground reaction force estimator based on IMU data.

## 1. Introduction

Measuring three-dimensional ground reaction forces (GRF), moments (GRM) and centre of pressure (CoP), as well as other biomechanical parameters, is a topic of great interest for the functional evaluation and biomechanics studies. The most common clinical exam, the Gait Analysis, requires the measurement of the walking kinematics and its boundary conditions represented by GRF, GRM and CoP [1]. Studying the GRF during sportive tasks, such as running, is important as many lower limb injuries have been associated with “overuse phenomena” resulting from the repeated impact loading of the foot [2,3]. The running or sportive performance may also be influenced by the type of surface on which it takes place, as it affects the load distribution. E.g. higher peak pressures were observed on asphalt at the central and lateral rearfoot while in the case of natural grass contact time and contact area were significantly greater at the central rearfoot [4]. Measuring GRF in sportive trials such as jumping or running is not an easy task, as the absolute value of recorded force may be as great as ~3–5-times the body weight (BW) [2,5]. Therefore, measurement protocols and sensors should be carefully designed to respect such ranges.

The state of the art method to measure biomechanical parameters in common activities, like walking or running, is by using an optoelectronic system (OS) in conjunction with two or more floor mounted force plates (FP). Such systems can be easily integrated with other acquisition devices such as electromyography, video recording or force sensors, providing reliable sets of data for an integrated, multifactorial functional evaluation [1]. Such systems were successfully applied to gait analysis [6], jumping analysis [7], upper limb evaluation and other physical tasks [8,9]. When repeated cyclic trials are required, e.g., to record several steps in walking or running, an instrumented treadmill can be used in conjunction with the OS [10,11]. The instrumented treadmill is equipped with force and pressure sensors that are able to accurately and directly measure the GRF and GRM, as well as the CoP, while the subject performs a cyclic task i.e., walking or running. In the most recent applications, the instrumented treadmill was integrated with virtual reality environment, involving a scenery dynamically evolving according to the pace and speed of the subject [11].

Despite its high accuracy, reliability, repeatability and its excellent metrological properties, the use of an instrumented treadmill in conjunction with an OS and a motion analysis laboratory has several drawbacks:

- It is inherently cumbersome and requires dedicated spaces and controlled environment, i.e., a motion analysis laboratory.
- It does not allow the measurement of tasks in open-field or requiring large spaces.
- It is expensive.
- It requires highly skilled operators.
- It was observed that subjects may change their walking strategy when walking on a treadmill instead of overground or open field [12,13].

In order to overcome the need for a laboratory environment, several techniques were developed to measure GRF, GRM and CoP by means of wearable sensors [14]. Some examples include shoe insoles made of a thin layer of strain gauge transducer [15], piezoelectric copolymer film [16], or an instrumented shoe equipped with force sensors beneath the forefoot and rear foot [17,18]. The instrumented shoe was proved able to measure the complete shear and vertical ground reaction force but the thickness of the sensor separated the shoe from the ground having an effect on: (i) the walking conditions; (ii) the friction between the walking surfaces; (iii) the height and weight of the effective sole [19].

Methods based on wearable sensors can be classified according to these categories:

- Methods based on matrix and/or pressure sensors used as insoles.
- Methods based on wearable load cells that directly measure three-dimensional GRF.
- Methods based on the kinematic data obtained by OS.
- Methods based on IMUs that measure motion of body segments and estimate GRF by means of a biomechanical model and/or machine learning methods.

The first three classes of methods were thoroughly discussed in the works of Abdul Razak et al. [14] and Shahabpoor & Pavic [20], therefore those topics go beyond the scope of the present review and the reader should refer to those papers [14,20]. To the best of author’s knowledge, no accurate review on the last class of methods currently exist in the literature. As the demand of wearable and minimally invasive sensors has dramatically grown in the last few years as they may provide valuable information for the functional evaluation of athletes in the sport fields. Therefore a comprehensive review of the methods for estimating ground reaction forces from inertial measurements represents a useful and needed support to both engineering and clinical research in the field. IMU based methods to estimate GRF are discussed in the present review.

The use of IMU experienced tremendous advances and became prominent in the last years thanks to the development of small and wearable sensors capable of recording accelerations, angular velocity and magnetic field [21]. Current hardware technology allows the capture and storage of a large volume of raw data and has inspired new paradigms of movement data interpretation [22]. Examples are the activity trackers that can be worn as bracelets and were proved able to track and identify the most common day’s activities and exercise levels [23]. Trackers were also used for the long term monitoring of physical activity in elderly population allowing to compute some health indicators such as energy expenditure, posture transitions, fall detection and balance analysis [24]. Most of the proposed methods require modelling of the biomechanical system to a certain extent. Such modelling in turn requires extensive knowledge of subject-specific parameters such as masses, dimensions, moment of inertia, etc. This inevitably introduces inaccuracies and uncertainty; therefore, these methods are currently the subject of much research. Nevertheless, there are several benefits in using IMUs to compute GRF as they are cost effective, easy to use and accessible to the general population.

This review is focussed on the methods designed in such a way to take advantage of wearable IMUs to indirectly estimate GRF, GRM and CoP during common activities such as walking or running.

## 2. Data Analysis

The systematic review was conducted on the basis of the PRISMA statement for conducting and reporting reviews [25]. This includes a pre-planned data analysis and pre-defined inclusion and exclusion criteria.

### 2.1. Search Strategy

Scopus, IEEE Xplore, MEDLINE and PubMed databases were interrogated on the topic of Ground Reaction Forces and Moments estimation based on kinematic data obtained by IMUs. Research keys included: ground reaction force and moment, vertical ground reaction force, inertial motion capture, accelerometers, IMU, inverse dynamics, gait analysis, running analysis, jump analysis, and similar. The latest database search was conducted in May 2018. Further references were identified by means of citations within the examined papers. Duplicate findings were removed. The records were then screened for potential inclusion.

### 2.2. Inclusion/Exclusion Criteria

**Figure 1:**
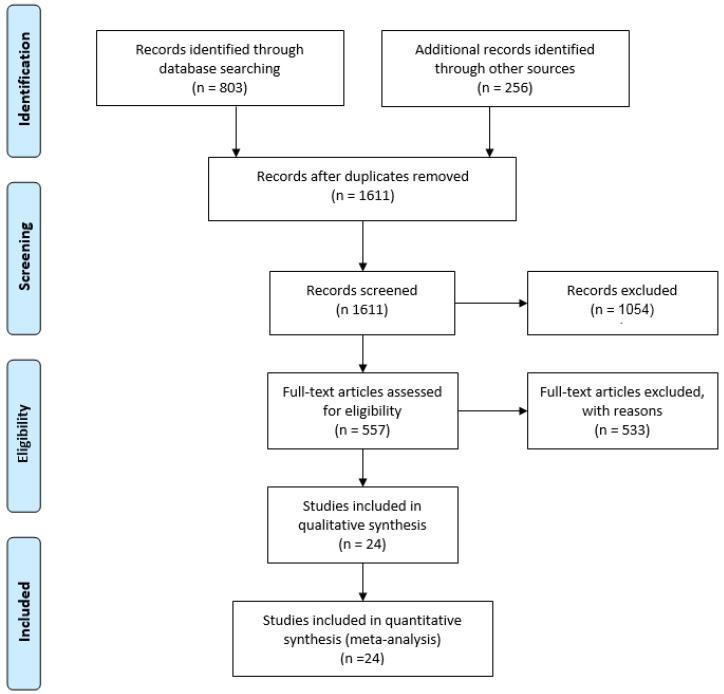
Study selection through the different phases using PRISMA framework [25].

The articles found through searches were first evaluated by title, keywords and abstract. Only English language peer-reviewed papers were included in the study. Conference abstracts and short articles were included only if they were found to provide relevant contribution. Eligibility criteria included: (i) articles proposing and/or validating methods for the estimation of GRF by means of inertial sensors; (ii) a well stated research question; (iii) appropriate statistical analysis; (iv) robust and repeatable data processing methods. Studies proposing measurement methods that cannot be used outside a laboratory or that relies on not-wearable technology, such as optoelectronic systems, treadmills etc. were excluded. Studies involving wearable instruments other than IMUs, such as insole pressure sensing, force sensors, etc. used as principal measurement device were excluded from the present review. Instead, the studies involving optoelectronic systems, pressure insoles or other devices for validation purposes were included. At the end of the screening process 24 papers were included in this study. Information from each article were organized in a pre-designed table, containing information on: type of sensor used, sensor placement, subjects involved, method used and general remarks/findings. The paper selection workflow is depicted in Figure 1.

The collected papers were organized in groups depending on the principal motor tasks that was discussed. We operated this division since the estimation of GRF may be significantly different depending on the task and support conditions required by that task (single support, double support, repeated contacts, etc.). Furthermore, papers were discussed according to the year of publication.

## 3. Discussion

### 3.1. Methods Based on Biomechanical Modelling

In order to estimate GRF from inertial data, some data modelling is needed. Most of the methods proposed in the earliest literature are based on inverse dynamics approaches that require biomechanical modelling. The methods examined were divided according to the task target of analysis.

#### 3.1.1. Walking and Running

**Figure 2:**
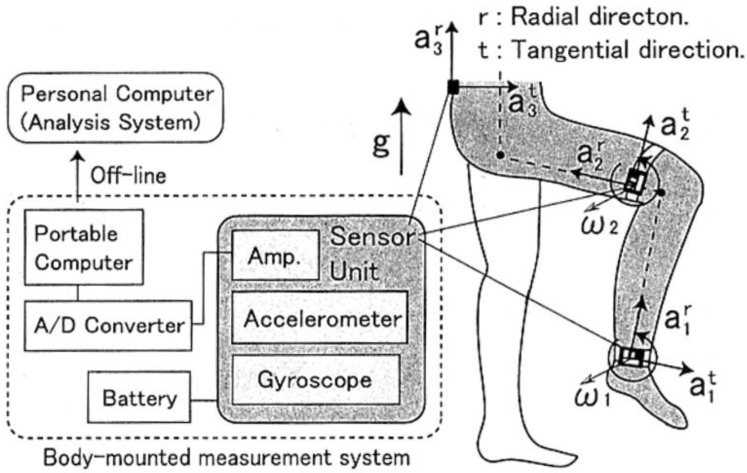
Accelerometer positioning and biomechanical model as designed by [26].

A first attempt to record kinematics and kinetics of human locomotion outside of a laboratory is the one by Ohtaki et al. [26]. They used three inertial units attached to distal position of shank and thigh with velcro straps (Figure 2) for long-term ambulatory monitoring. The adopted configuration allowed to study only the kinematics on the sagittal plane. Moreover, only the motion of the left leg was measured and the kinematics of the right leg was obtained under the assumption of left-right symmetry in normal gait. Measuring the single leg motion allowed to: (i) eliminate sensors on the other leg improving mobility; (ii) reduce power consumption. Each inertial unit was composed of a uni-axial accelerometer (range ±5 g) and uni-axial gyroscope (range ±300 deg/s). The data was logged on a notebook computer carried in a backpack while subjects walked on a straight line at a pace defined by a metronome. The estimated results were compared to the data collected by an OS and FP.

The temporal gait parameters and the kinematics could be derived by means of some dedicated algorithm based on the frequency components of acceleration. High frequency values were associated to heel strike (HS) events while lower frequencies were associated to voluntary body movements or inclination with respect to the gravity direction. Signals were low-pass-filtered at 10 Hz and high-pass filtered at 30 Hz. Heel strike events were identified by spikes in high frequency components while the derivative of the angular velocity was used to identify false detections, as the derivative of velocity is always negative around the true heel contact instance [26].

**Figure 3:**
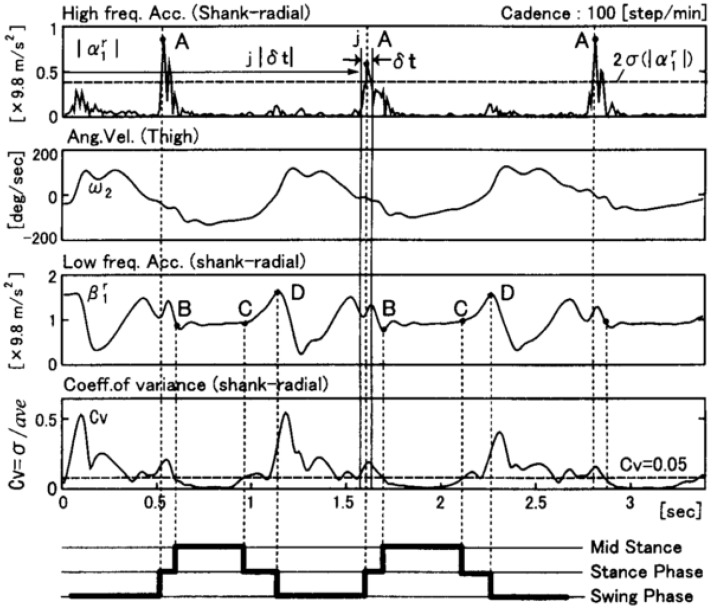
Detection of walking phases from the radial acceleration of the shank according to the algorithm proposed by [26]. A: heel strike, B: beginning of mid stance, C: rising of heel, D: toe off.

The algorithm was also capable of identifying mid-stance and terminal stance of single support phase, by detecting recurrent patterns in the radial acceleration of the shank. The detection of those phases is illustrated in Figure 3.

Joint angles were calculated by integrating angular velocities of the five-segments composing the body model and taking advantage of standard anthropometric data [27]. The model is depicted in Figure 2 and it is composed of five segments: pelvis, thighs and shanks. The total ground reaction force on each leg was determined by an inverse dynamic analysis based on the recursive formulation of force and moment balance equations. During single support stance phase the total GRF was estimated as the sum of forces on each of the five segments of the model (Equation (1)). The acceleration of the left leg was estimated assuming bilateral symmetry (Equation (2)).

(1) $$F^{stance}=\overset{5}{\underset{i=1}{\sum }}m_{i}*a_{i}\left(t\right),\;\;\;\;t\in mid\;stance$$

(2) $$a_{left}\left(t\right)=a_{right}\left(t+T/2\right)$$

where *m_i_* and *a_i_* are the mass and acceleration of the *i*-th segment of the model. *T* is the stance time. The acceleration was computed at the centre of mass of each segment by rotating and translating the acceleration measured.

Joint moment $\tau_{i}$ was computed by solving the equations of motion and the power was computed by multiplying moment and angular velocity $\omega_{i}$ as in Equation (3):

(3) $$P_{i}=\left|\tau_{i}*\omega_{i}\right|$$

This method proved able to detect temporal parameters according to foot movement, kinematic data and ground reaction forces [26] although some deviation was observed when compared to the OS. Maximum RMSE was 11.2° for angles and 0.31 N/BW for GRF (the GRF were normalized to bodyweight). However, several limitations were observed. First of all, it used a much simplified model of the human body and standard anatomical parameters. Only one-dimensional sensors were used and the analysis was limited to the sagittal plane. This introduced inaccuracies in the estimation of kinematic parameters that propagated to the computation of forces. Moreover the analysis was limited to the single support phase. In double support phase, when both feet are touching the ground, the kinematic chain is indeterminate and the equations of motion cannot be solved [20]. Other sources of error were attributed to the soft-tissue artefacts due to sensor fixation [26]. While proposing a valid approach to the estimation of GRF, this method had some serious limitation that were partially overcome by the subsequent studies.

A different approach was the one proposed by Neugebauer et al. [28] that used a statistically based model to estimate the peak of the vertical component of GRF during walking and running. Data were recorded by means of a low-cost bi-axial accelerometer, allowing a sampling frequency of 40 Hz and measurement range of ±7 g. The sensor was fixed over the most lateral aspect of the iliac crest of the right hip and it measured the maximum acceleration recorded on the two axes. The estimation of the peak ground reaction force during the support phases was based on a statistical model based on repeated measures and a mixed effects regression. Such model looks for a linear or logarithmic relation between two variables by taking into account the effects of several different factors expressed in a hierarchical form [29]. In this case, the model was based on the assumption that sex, body mass and type of locomotion were good predicting factors of the relationship between acceleration and GRF. This assumption was based on the fundamental equations of motion where the GRF is function of mass, inertial properties of body segments and acceleration of body parts [28]. Data analysis demonstrated that the body mass was indeed a good predictor factor and the logarithmically transformed peak GRF was well predicted using the mixed effect model. The average absolute difference between predicted GRF and the one directly measured by the force plate was 9% while the maximum observed error was 17.5% [28]. However, this approach had several limitations, mainly due to the sensor used. The type of sensor and its positioning did not allow the measurement of spatio-temporal parameters as well as the temporal profiles of the GRF. Furthermore, the method used a statistical approach rather than a more detailed biomechanical model, therefore many anatomical features of the subjects were not taken into account. Thus, the method may not be suitable for activities involving concentrated and repeated loads, such as in jumping or during a training session. The same authors further explored this method in a subsequent work [30]. They recorded accelerations using a 3-axis accelerometer located, again, at the most lateral aspect of the waist, over the right iliac crest. This new device was able to record data at a sampling frequency of 100 Hz, and the maximum range was ±6 g. The aim of the work was to compute peak GRF from the acceleration recorded at the hip by using a mathematical model based on linear regression [30]. The authors found that acceleration measured using a hip-mounted accelerometer may not provide an accurate representation of the load sustained by the body. In fact the developed model was found to underestimate the peak GRF, especially in those tasks associated to higher peak GRF, such as running. Moreover, the authors observed that in some cases the peak acceleration at the hip, while running, could be higher than ±11 g [30] which saturated the accelerometer range of ±6 g. Thus, this approach should be used cautiously and other accelerometer configurations have to be preferred.

Another method to estimate peak GRF in running is the one by Wundersitz et al. [31] that used only a tri-axial accelerometer fixed on the upper body. The fundamental hypothesis was that peak GRF in running is caused by the collision of the foot with the ground [32] and, being the mass constant, the measured acceleration is proportional to force [33]. The sensor was placed on upper body as in previous studies it was stressed that the sensor should be placed in such a way to interfere as little as possible with an athlete’s performance [34,35]. The use of a 3-axis accelerometer, instead of a 1-axis one, provided increased sensitivity to the impact acceleration due to increased cross-axis sensitivity [31]. The IMU used in [31] was composed by a 3-axis accelerometer, range ±8 g, sampling frequency 100 Hz. The sensor was fixed in the centre of the upper back at the level of the second thoracic vertebrae as in previous studies [36]. The main axis of the accelerometer was along the crania-caudal direction and close to equivalent with the global vertical axis [31]. The peak GRF estimated by this method was compared to the output of a force plate while the subjects performed several running tasks and direction changing tasks [31]. Smoothing the acceleration signal was proved essential to obtain reliable data and suggested that the optimal low-pass frequency was 10 Hz. With low-pass, the peak GRF estimated by the accelerometer was comparable to the one directly measured by the force plate. Over multiple trials, accelerometers may provide an acceptable measure of impact force. The absolute error for a single measurement was ~24%. Thus data smoothing was recommended.

A similar approach was proposed by Charry et al. [37] that used a three-axis accelerometer fixed on the medial tibia of each leg. The tibial acceleration was measured while running. This approach was based on a previous work where tibial shock was quantified by taking advance of a linear relationship between the tibial axial acceleration and the peak GRF [38]. The two commercial accelerometers had range ±24 g, sampling frequency 100 Hz, and were fixed on both tibias, along the tibial axis in the midpoint between the lower edge of the medial malleolus and the medial joint line of the knee. The direct measurement of the GRF by a force platform was used for comparison.

From the recorded tibial acceleration profile, it was possible to identify four events: (i) heel strike; (ii) Initial Peak Acceleration; (iii) Maximum Peak and (iv) Peak to Peak acceleration. The authors found that a logarithmic fitting would best approximate the correlation between acceleration and peak GRF. This method could then be exploited to identify walking phases from the acceleration signal. The RMSE error in the logarithmic estimation of GRF from acceleration, compared to the direct measurement from the force plate, reached an average of ~150 N across different running speeds [37].

Meyer et al. [39] studied the validity of the accelerometer based method to measure GRF in children. The tasks performed were walking, jogging, running, landing from boxes with heights of 10, 20 and 30 cm, rope skipping and dancing some breakdance moves. Ground reaction forces were simultaneously recorded by force plates. During the tests, the children wore two different commercially available tri-axial accelerometers at their right hip. Sampling frequency was 100 Hz and maximum range ±8 g and ±6 g, respectively. The GRF measured by means of the FP were: 1.3 times the BW for walking, 2.2 BW for jogging, 2.8 BW for running. In case of landing from different heights, the measured forces were: 4.2 BW for 10 cm, 5.2 BW for 20 cm, 5.9 BW for 30 cm. The correlation between the FP and the measured accelerations was very high (R = 0.90). Sex, age, weight, height and leg length of the children did not have a significant influence on the correlation coefficients. Despite the high correlation between the applied methods, both accelerometers systematically overestimated the GRF and the measurement bias increased with loading [39]. Although accelerometer data had a good correlation with measured GRF, the authors recommended caution in using accelerometers when an absolute measurement of force is required. The authors also stressed the importance of using an adequate sampling frequency that should be at least twice the speed of the fastest movement [40]. The frequencies for normal non-impact physical activities in humans are generally below 8 Hz [41] but during peak contacts (e.g., in running) frequencies may be higher [39]. Maximum ranges of ±8 g and ±6 g may also be limiting for very high impact loadings, while values ranging from 2 g to 4 g were observed in common tasks. Such values of acceleration were proved sufficient to induce beneficial structural changes in bone strength [42,43].

**Figure 4:**
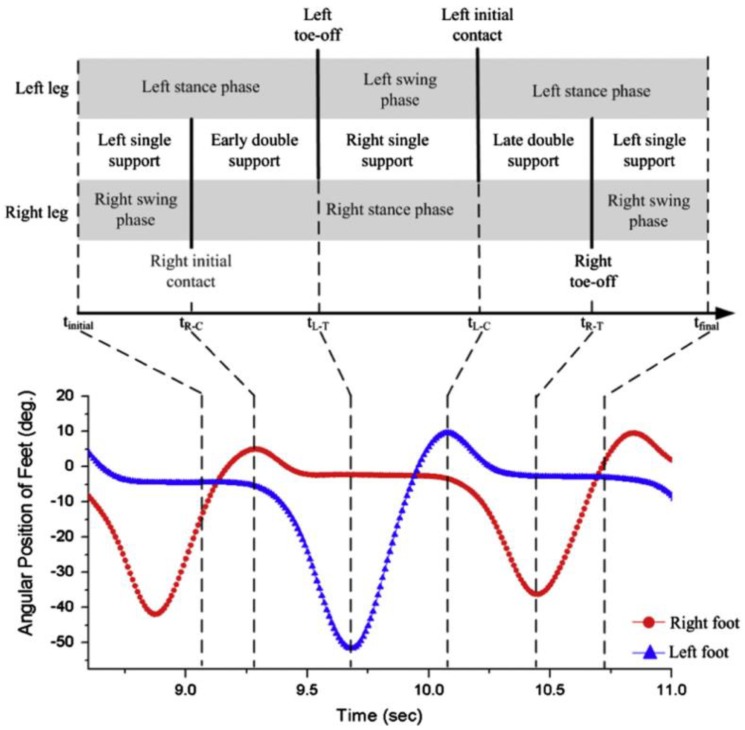
Identification of gait phases from the angular position of the feet, according to [44].

A more complex approach is the one proposed by Yang et al. [44] that designed a method to estimate lower limb forces and moments while walking by using a kinematic tracking device. This method was aimed to be used for the clinical analysis of walking in clinical environments without the need of a dedicated laboratory or expensive instrumentation. The study targeted a walking task as it is the most common activity being object of clinical studies in people with motor injuries. The authors placed gyroscopes at the mass centres of the trunk, thighs, shanks and feet in order to measure the corresponding angular velocities, while accelerometers were placed on the feet to measure their linear acceleration. As an advancement from previous methods, this one aimed to measure three-dimensional walking motion, meaning that kinematics in frontal and transverse planes were considered in addition to the sagittal plane. Since the aim of the work was to estimate not only the GRF but also the intersegmental forces on the lower limb, a detailed biomechanical model was needed. Mechanical properties (mass and inertial moment) were assigned to each segment of the model based on standard values reported in the literature [44]. The angular positions and acceleration of the hips, knees, and ankles were obtained by integrating and differentiating the measured angular velocities. The walking cycle was identified and segmented from the angular position of the feet (Figure 4). This analysis allowed the identification of leg support conditions: (i) early double support; (ii) single support; (iii) late double support. These phases were identified from the instants of initial contact and toe-off which in turn were identified directly from the IMU [44].

By taking advantage of the IMUs placed on body segments and knowing the mass of each segment, the force of each intersegmental joint was computed. Starting from the hip, the forces on each lower limb joint were subsequently computed by summing the load found on the upper segment as shown in Equations (4)–(8). The last forces to be computed were the one on the heel and the one on the phalange. The force on the other hip, namely $f_{L-\mathrm{hip}\;}$, was estimated by means of an exponential transfer function [44].

(4) $$f_{R-\mathrm{hip}}=m_{\mathrm{trunk}}\left(a_{\mathrm{trunk}}-g\right)-f_{L-\mathrm{hip}}$$

(5) $$f_{R-\mathrm{knee}}=f_{R-\mathrm{hip}}+m_{\mathrm{thigh}}\left(a_{\mathrm{thigh}}-g\right)$$

(6) $$f_{R-\mathrm{ankle}}=f_{R-\mathrm{knee}}+m_{\mathrm{shank}}\left(a_{\mathrm{shank}}-g\right)$$

(7) $$f_{R-\mathrm{foot}}=f_{R-\mathrm{heel}}+f_{R-\mathrm{ph}}=f_{R-\mathrm{ankle}}+m_{\mathrm{foot}}\left(a_{\mathrm{foot}}-g\right)$$

(8) $$f_{R-\mathrm{ph}}=sf_{R-\mathrm{foot}}\;\mathrm{an}\;f_{R-\mathrm{heel}}=\left(1-s\right)f_{R-\mathrm{foot}}\;\mathrm{where}\;s=\frac{l_{\mathrm{PC}}}{l_{\mathrm{foot}}}$$

where $l_{\mathrm{PC}}$ is the distance between the pressure centre and the heel, $l_{\mathrm{foot}}$ is the length of the foot.

**Figure 5:**
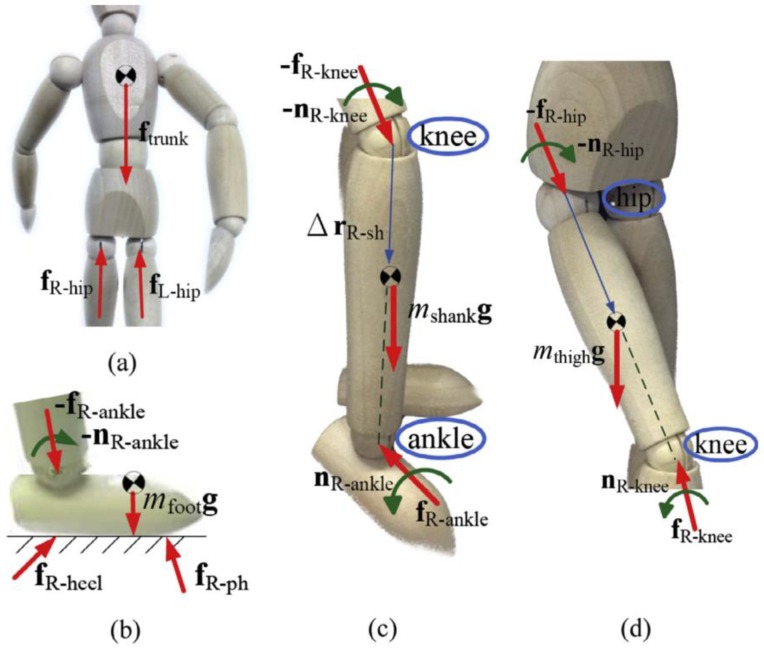
Biomechanical model as defined by [44] and the respective free body diagrams of: (**a**) trunk, (**b**) foot, (**c**) lower leg, (**d**) upper leg.

**Figure 6:**
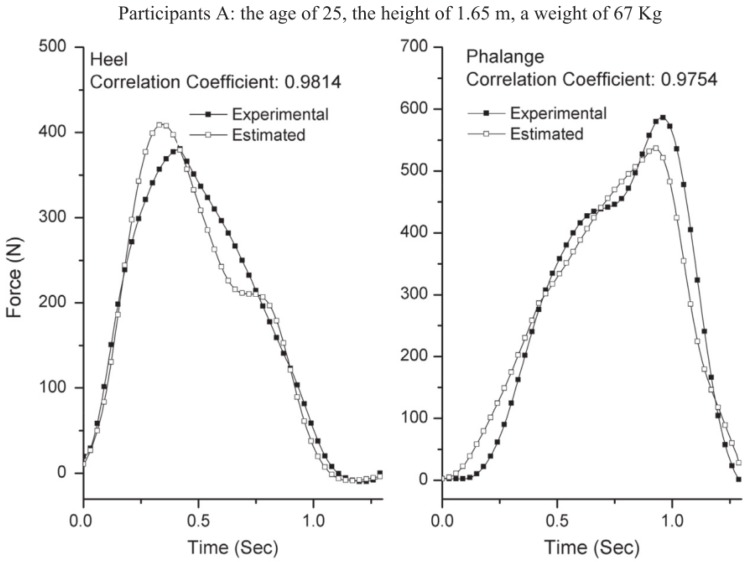
Forces on heel and phalange estimated by the method proposed by [44] and compared to the output of load cells placed under the shoe.

The forces on heel and phalange estimated by this method were compared to the forces measured by means of load cells placed under the shoe. The biomechanical model is shown in Figure 5. Estimated forces were found in good agreement with the measured ones (Figure 6), a good correlation was observed between the two signals (R > 0.95) and a relatively low maximum RMSE of ~66 N.

The method proposed by [44] was more complex than previous ones as it used seven IMUs and an enhanced three-dimensional biomechanical model, but it enabled the maximum GRF to be evaluated without the need of force plates and the intersegmental forces to be estimated without having to use invasive sensors. However, the weakness of this approach was in the estimation of forces during the double-support phase as the distribution of the force across the two legs was evaluated through a statistical approach.

**Figure 7:**
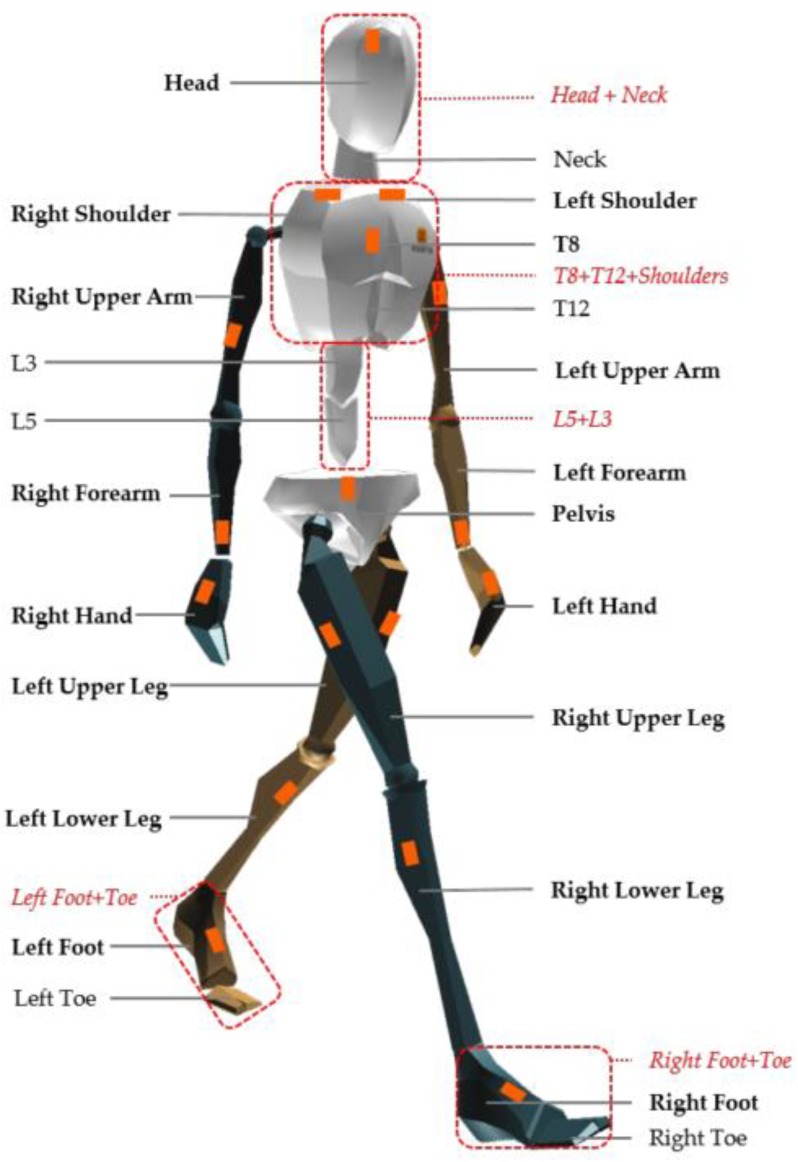
Biomechanical model and landmarks for IMUs as proposed by [45].

Karatsidis et al. [45] also designed a method to predict both GRF and GRM during walking using only kinematic data from IMUs and they tried to overcome the indeterminacy problem in double support phase by using a distribution algorithm based on a smooth transition assumption. The authors used an inertial system composed of 17 IMUs mounted on a fitting suit whose sensor landmarks are shown in Figure 7. Sampling frequency was 240 Hz. The output of the IMUs was comparatively examined to the output of an OS and FP. The kinematics of the 23 anatomical segments composing the model was reconstructed by taking advantage of the acceleration signals acquired by the IMUs. From the kinematics and inertial properties of each segment, the total external force was estimated from Newton’s equation of motion, Equation (9), [46]. Similarly, the total external moment was computed from Euler’s equation, (Equation (10)).

(9) $$F_{ext}=\overset{N}{\underset{i=1}{\sum }}m_{i}\left(a_{i}-g\right)$$

(10) $$M_{ext}=\;\overset{N}{\underset{i=1}{\sum }}\left[J_{i}{\dot{\omega }}_{i}+\omega_{i}\times \left(J_{i}\omega_{i}\right)\right]-\overset{N}{\underset{i=1}{\sum }}\overset{K_{i}}{\underset{j=1}{\sum }}\left(r_{ij}\times F_{ij}\right)$$

where $K_{i}$ is the number of endpoints in each segment, $\omega_{i}$ is the angular velocity of the *i*-th segment, $J_{i}$ is the inertia tensor around the centre of mass of the *i*-th segment, $r_{ij}$ is the lever arm between the centre of mass and the applied force $F_{ij}$.

**Figure 8:**
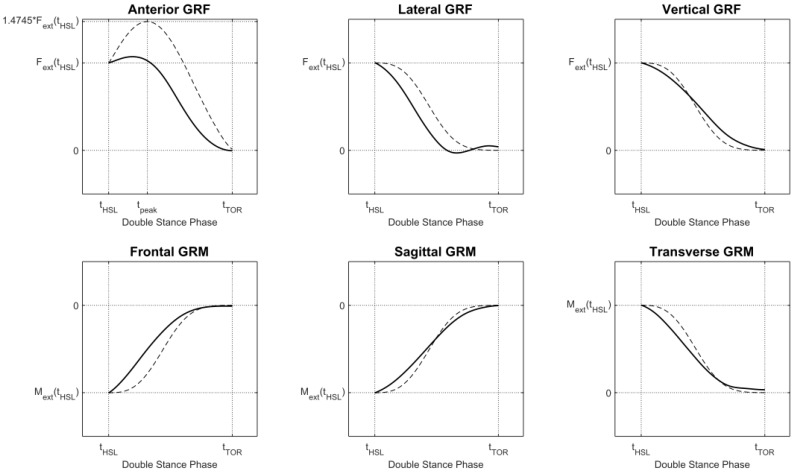
Curves for smooth transition assumption used to distribute external forces and moments among the two feet [45]. Curves were built from empirical data. Dashed lines represents the curves as obtained in a previous study [47].

The inertial parameters of each segment were computed through scaled anthropometric data as suggested in [27]. During the single support phase the GRF was computed as in previous methods, while GRM was computed assuming the lever arm of the applied GRF as the projection of the ankle to the ground. During double support, the solution to Newton’s equation is indeterminate, thus the authors implemented a distribution algorithm based on a smooth transition assumption function built on empirical data [45]. The function, illustrated in Figure 8, depends on the timings of gait events and was used to distribute force and moments among the two feet during the double support phase.

The phases of single and double support were identified by means of a gait event detection algorithm. The procedure was based on a threshold level applied to the norm of the velocities of heel and toe [45]. The estimated GRF and GRM were compared to the ones measured by means of OS and FP throughout three sub-phases of the walking cycle: (i) first double support; (ii) second double support; (iii) single support of each foot.

It was found that inertial motion capture and optical motion capture systems had similar performance in estimating GRF and GRM when compared to the gold standard force plates. The highest RMSE errors for GRF were observed for the lateral component of force. Worst results were the estimation of lateral force in a “fast walking” task to which corresponded a RMSE of 14.6%. The maximum RMSE for GRM was 30.6% and it was observed for the frontal force in a “fast walking” task [45]. Regarding the stride phases, the highest errors were observed in the double support phases.

In general, the anterior and vertical GRF, as well as the sagittal GRM estimates performed better than the lateral GRF and frontal and transverse GRM. This was explained by the smaller magnitude of the lateral measures that have a relatively large impact on the final estimates.

This method had some limitations. First, the estimation of GRF during double support had poor accuracy due to the fact that the smooth transition assumption was based on empirically derived curves obtained from healthy subjects, thus this method is not suitable for people with movement disorders. Second, this method may not be accurate for slower or faster walking speeds, running or abnormal walking where a more sophisticated force distribution model is required. Third, the mechanical properties of each segment were based on average anthropometric data that may not represent correctly elderly or obese populations [48,49]. Finally, there are some intrinsic issues in the use of inertial sensors mainly due to magnetic interferences and soft tissue artifacts. This method was based on a 17 IMUs full body protocol and reducing the number of sensors may make the system more practical for clinical and use in sports.

Estimating GRF during double support represents the most critical challenge when one can rely only on kinematic data. In fact, during double support the lower limbs form a closed loop mechanical chain, making it impossible to uniquely determine GRF at each foot by relying only on Newton-Euler equations. The method proposed to work around this was the “smooth transition assumption” [45] as well as other previously developed mathematical models to predict the transition of the load from the trailing to the leading leg [50,51,52]. Such methods use statistical models, mainly based on empirical data, to predict the amount of load to be assigned to each foot across the double support phases. Another method is the one proposed by Dijkstra et al. [53] that exploited the “Zero Moment Point” i.e., the point on the ground at which the horizontal moments due to external loads are null. In stability conditions, this point coincides with the centre of pressure. This method is computationally inexpensive and it is commonly used to stabilize the bipedal walking of robots [54]. The “Zero Moment Point” method was tested on kinematics data obtained from an optoelectronic system, force platforms and an OpenSim [55] body model. It was proved that that the estimated GRF were accurate in the vertical and lateral directions while the forces on the anterior posterior direction were underestimated, spreading also inaccuracies to the estimation of joint moments [53].

**Figure 9:**
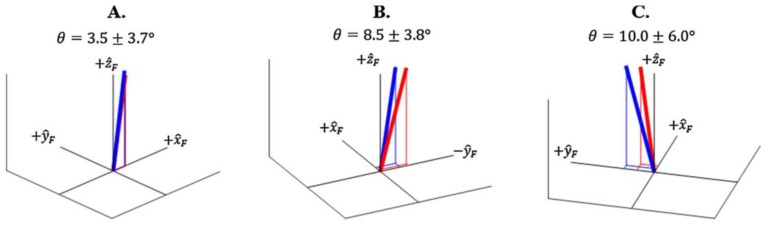
Graphical representation of the GRF vector estimated by the IMU (red) and by the force platform (blue) as found by [56]. (**A**) sprint start task, (**B**,**C**) change of direction tasks. Angular error between the vectors is represented.

**Figure 10:**
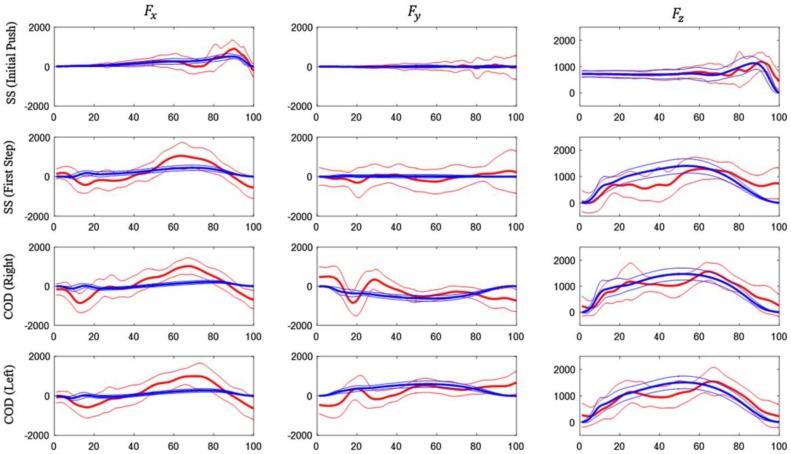
Graphical representation of the GRF curves along the gait cycle for IMU (red) and FP (blue) as found by [56]. (SS) sprint start task, (COD) change of direction tasks. F_z_ is the vertical component.

Gurchiek et al. [56] conducted a feasibility study regarding the use of a single IMU placed on the sacrum. The IMU was placed close to the centre of mass in order to measure translational acceleration at this point. As in previous studies, the total force was estimated by means of a simple model based on Newton’s law [31,57]. In addition to this, the authors used the information from the gyroscope and magnetometer to estimate the orientation of the body segment, allowing the expression of the sensor referenced vectors in an inertial referenced frame. The three-dimensional force estimated in this way was compared to the measurements of a force plate. The subjects performed acceleration and change of direction tasks [56]. Two static calibration trials were needed in order to reconstruct the position of IMU with respect of the ground reference system. Information from the IMU magnetometer and accelerometer were used to estimate the initial heading. Then, the measured quantities were referenced to the ground reference system by means of quaternion math [58]. A good agreement was obtained between the vertical component of force estimated by the IMU and the one measured by the FP (Figure 9 and Figure 10). However, a poor agreement was observed in the case of medio-lateral and antero-posterior components (Figure 9 and Figure 10). Thus, this method could be recommended only for the estimation of the vertical component of GRF and its magnitude. In terms of three-dimensional force vectors, the maximum angular error observed between the vectors estimated by the IMU and the FP was 10° [56].

This approach is very interesting as the use of a single IMU sensor dramatically simplifies clinical measurements by means of wearable sensors. However, according to [56], it was reliable only for the estimation of GRF in the sagittal plane. There are two other major limitations: firstly, the actual center of mass changes its position, with respect of the IMU on the sacrum, during activities such as walking or running. Pelvic displacements and rotations may therefore induce artifacts in the estimation of forces [56]. Secondly, the use of magnetometers to estimate IMU orientation is affected by ferromagnetic disturbances that can lead to reduced accuracy of results.

Raper et al. [59] designed a protocol to measure GRF by means of a single IMU mounted on the mid portion of the medial tibia and conducted a reliability analysis of this protocol by comparing the result to a force platform. The IMU was composed of a tri-axial accelerometer with sampling frequency of 100 Hz. Analysis was conducted by means of the software provided by the manufacturer that provides the calculation of the peak GRF from the vertical component of tibial acceleration. The subjects were professional athletes that were asked to run indoor on a track equipped with piezoelectric FPs. Each foot contact was identified and computed GRF was matched to the one directly measured by the FP. The absolute value of the GRF as measured by the IMU was different from the one recorded by the FP. It was observed that the IMU could underestimate the force up to 400 N [59]. This error was supposed due to a delay between the peak in acceleration and the peak of exerted force. The authors recommended that the IMU measurement should not be interchanged with the Newton unit of measurement, but it is yet capable of measuring lower limb load in running tasks. The accuracy was estimated as high as 83.96% and the reliability was very high with an ICC of 0.97, thus the IMU could be considered an useful tool for measuring lower limb load in athletes performing sportive tasks [59].

**Figure 11:**
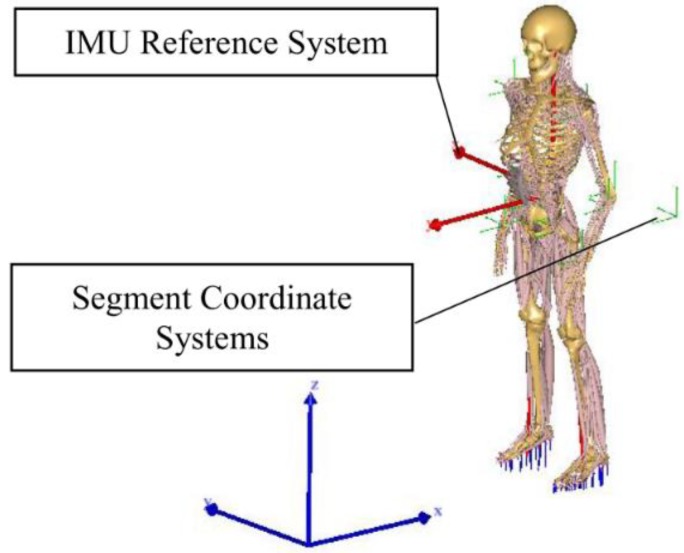
Musculoskeletal model designed by [60]. The coordinate frame within the hip represents the reference system for the IMUs.

**Figure 12:**
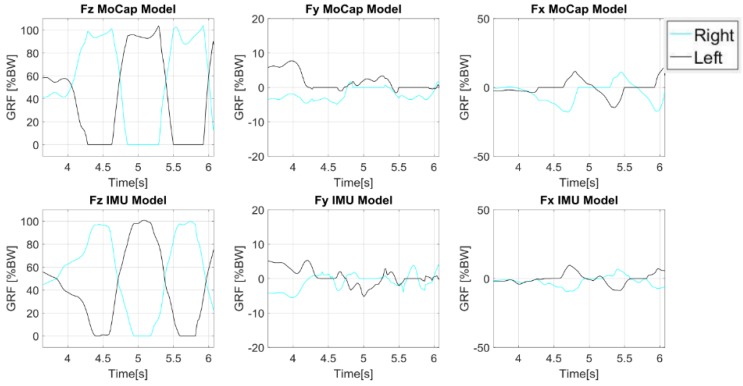
Components of Ground Reaction Forces estimated from the model designed by [60]. On the first row: forces estimated by the OS, on the second row: forces estimated by IMUs.

A more advanced anatomical model is the one used by Aurbach et al. [60] that implemented a musculoskeletal model (Figure 11) taking advantage of the software AnyBody™ (AnyBody Technology A/S, Aalborg, Denmark). The model could rely on a detailed anatomical representation of the skeleton and on an inverse kinetic engine to reconstruct forces from the measured kinematics. The model could compute the GRF as well as the forces acting on the ankle, knee and hip. Kinematic data was collected by means of 15 IMUs mounted on: the top side of the feet, the anterior side of the shanks, the anterior side of the thighs, one at the sacrum, one between the shoulder blades on the back and one at the forehead. Calibration data, obtained during the initial stance phase, was needed to align the magnetometers and accelerometers to the local reference system. The standing humanoid model was translationally fixed at the hip segment, as the IMU system may only provide rotational information. The model was solved two times by taking advantage of the kinematics concurrently recorded by: (i) an OS; (ii) the IMUs. Results were then compared. The comparison is shown in Figure 12. The OS data displayed a longer period of one sided loading of the feet and a difference in curve progression [60].

This study showed that the IMU based model yields the possibility to estimate the GRF independently of gait labs, however IMU performance was poor when compared to the OS [60]. The main sources of errors were attributed to the fluctuations within the magnetic field that could not completely be removed by calibration procedure. In fact, the IMUs used made extensive use of internal magnetometers to fully estimate their orientation in space and magnetometers are easily affected by electromagnetic disturbance or ferromagnetic objects nearby [61]. Another limitation of this approach lies within the configuration of the humanoid model that should match exactly the physical characteristics of the subject. Thus, an accurate subject calibration is required and further work is necessary to minimize the issues with the IMU model.

Thiel et al. [62] tested the GRF during sprint running by means of IMUs. They used two IMUs composed of 3D accelerometer, gyroscope and magnetometer. Sampling frequency was 250 Hz and data was logged locally. The IMUs were placed on the shank above the medial malleolus. The accelerometer data was aligned and compared to a force platform used as comparison, while the athletes were asked to run on an instrumented running track. The vertical component of GRF was assumed related to the shank acceleration by the following linear equation:

(11) $$F_{v}=c_{1}a_{x}+c_{2}a_{y}+c_{3}a_{z}$$

where *a_x,y,z_* are the components of measured acceleration and *c*_1,2,3_, are empirical coefficients.

**Figure 13:**
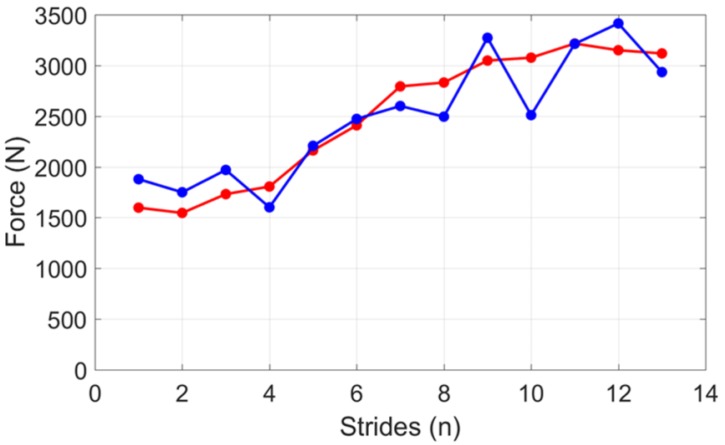
The vertical GRF measured by the force plate (red) compared to the one predicted by the IMUs (blue) for one subject, according to the method by [62].

The coefficients were determined for each foot by taking advantage of the force recorded in the first steps [62]. This kind of modelling was proved suitable for the first stages of the sprint where a constant GRF is expected and therefore a linear modelling is suitable [62]. In addition, the shank angular velocity was used to identify stance and swing phases and, as a consequence, to identify timings of the peak GRF. The authors found that this method was not reliable for every participant even though it could accurately predict the peak GRF for one subject (Figure 13). The potential sources of error were identified in the force attenuation at the ankle due to the musculoskeletal structure and the absorption of the running shoe. This effect may be attenuated by means of a calibration procedure.

The task of running was further explored by Kiernan et al. [63]. The aim of their study was to study micro traumas and injury mechanisms due to repeated loads occurring in runners. The magnitude of peak vertical GRF was estimated by means of a three-dimensional accelerometer worn on the right hip. Recording range was ±8 g and sampling frequency 48 Hz. Hip acceleration was recorded over an entire training session. The use of antero-posterior components of acceleration allowed to identify right and left foot strikes by means of a dedicated algorithm [64]. Only the right strides were considered and the peak GRF during stance was estimated by means of a linear regression model as in [30]. Then the mean of peak GRF was calculated among the considered strides. The number of strides was counted as well. The participants were separated into two groups: injured and non-injured. The comparison of those groups demonstrated that the injured subjects had higher peak vertical GRF values as well as cumulative loads. Thus, such a method is capable of predicting injuries and capture loading profiles of participants [63]. However further study is necessary to better assess the effects of repetitive loads, when the maximum load occurs during the training and most important, the effects of asymmetry in loading left and right legs. The method proposed by [63] was not able to assess such quantities, thus further studies and more advanced protocols are needed. The use of wearable IMU seems a promising method to measure such quantities in running.

#### 3.1.2. Jumping and Other Tasks

Jumping, squat and bending tasks are different from walking and running. First the GRF are always distributed among the two feet and single support rarely occurs, second the motion occurs mainly along the vertical axis and it is not cyclical. The indirect estimation of the GRF on each foot is difficult as well as the detection of asymmetry in foot loading.

An early work on the estimation of GRF in vertical jumping was the one by Elvin et al. [65] that supposed a correlation between the peak vertical GRF and the peak tibial vertical acceleration. The subjects were asked to jump at different heights while the shank acceleration was recorded. The acceleration was measured by means of two uniaxial accelerometers worn on a support sleeve placed close to the vestibular heads on both legs, while the GRF was measured by means of a FP. The landmarks for placing the accelerometers where chosen because, according to the authors: (i) it is easy to locate the landmark through palpation; (ii) the risk of the sensor causing an injury to the subject was low; and (iii) it has been previously used as an accelerometer attachment site [65]. The range of measurement was ±70 g and sampling frequency 1 kHz. The authors found that the peak GRF in landing could reach up to 8.2 times the body weight while the peak tibial acceleration could reach up to 42.3 g. A strong correlation was observed between peak GRF and peak acceleration (average *R*^2^ = 0.812, *p* ≤ 0.01), thus the authors concluded that the peak GRF may be computed from Newton’s second law by knowing subject’s mass. This study helped identifying several inaccuracies in the procedure such as: (i) the relative movement of the accelerometer with respect to the body; (ii) noise and non-removable instrumental errors; (iii) unmeasured angle between tibia and ground during impact; (iv) possible non-linearity in the relationship between acceleration and GRF [65]. Moreover, a clear correlation was not identified between the height of the jump and peak impact forces while it was demonstrated by other studies [7]. Interestingly, the authors were able to compute the flying time for the jump and hence the vertical height from the temporal profile of vertical acceleration by using a previously validated algorithm [66].

The task of vertical jump was further investigated by Howard et al. [67] by means of a tri-axial accelerometer placed close to the centre of mass. GRF were simultaneously measured by means of a FP while the subjects were performing some countermovement and drop jumps. Minimum eccentric force and peak concentric force were calculated concurrently for countermovement jumps and peak landing forces were calculated concurrently for drop jumps. The authors found a good agreement between the accelerometer and FP during the eccentric phase of the countermovement jump but a consistent systematic bias between the results from the force platform and accelerometer was observed. Therefore the force obtained from the measurement of acceleration could not be used interchangeably with the force measured by the FP. Thus, it was recommended to use gyroscopes to increase accuracy of datasets [67].

Pouliot-Laforte et al. [68] assessed the validity of GRF in vertical jumping tasks when estimated only by an accelerometer. The analysis was conducted on both healthy children and children diagnosed with Osteogenesis Imperfecta type I, a pathology that commonly gives rise to several functional limitations and muscular weakness [69,70]. In such cases, having a simple and portable measurement system would be highly valuable for the assessment of GRFs and thereof the mechanical loading of the bones. The estimation of the GRF was obtained by applying Newton’s law of motion, i.e., multiplying the mass of the subject times the measured vertical acceleration, as in previous studies. High intensity actions like jumps are known to generate high peak forces at a high rate. The subjects were asked to perform five different jump and some rise manoeuvres on a portable force platform while wearing an accelerometer on the right hip. The accelerometer recorded data at a sampling frequency of 60 Hz and had range of ±6 g. The estimated GRF was compared to the one recorded by a FP. The accelerometer was placed on the patient’s right waist slightly behind the anterior iliac crest and held in place by an elastic band. As in previous studies, the results showed a high correlation and good agreement between the GRF measured by the accelerometer and by the FP. Thus, measuring GRFs with an accelerometer is a potentially valuable tool to estimate ground reaction forces in children and adolescents, both healthy and with pathology [68]. The small over/underestimation of averaged GRFs by the accelerometer’s derived forces suggested that over long recordings force measurements are quite accurate. The authors concluded that, while placing the accelerometer on the right hip was extensively validated in the literature, it would be preferable to place the sensor near the centre of mass, i.e., the lower back.

The GRF during a squat motion was measured by Min et al. [71] that comparatively examined the IMU performance against a FP and OS measurements. Squat motion was modelled on the sagittal plane as a 3-segments linkage and motion was recorded by means of three IMUs placed on the lower back, thigh and shank. The local x-axes of the IMUs were aligned as normal to the sagittal plane. IMU sensors recorded 3-dimensional accelerations, angular velocities and magnetic fields. Sampling frequency was 100 Hz. The IMUs captured the kinematics of each segment of the model and reconstruct GRF through inverse kinetics. The computed GRF showed high accuracy when compared to FP (RMSE < 0.02 BW). However, the analysis and the biomechanical model were limited to the squat movement and other approaches may be necessary to investigate other tasks, such as running.

**Figure 14:**
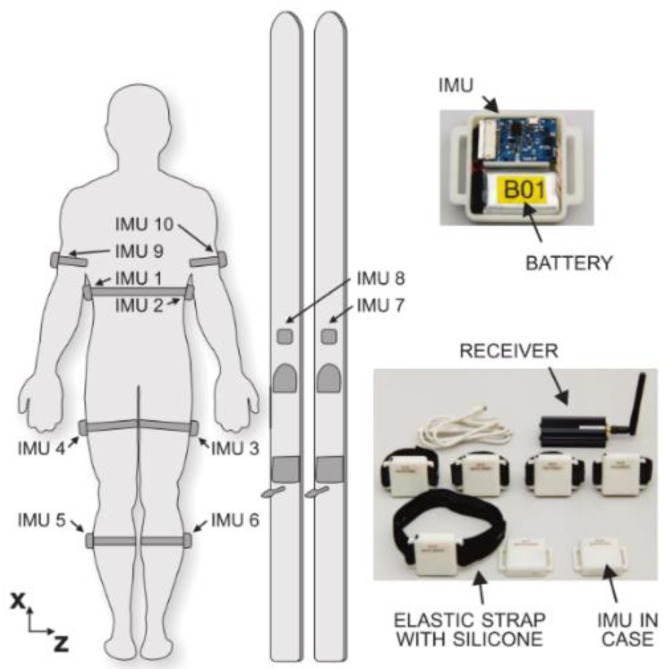
IMU placement landmarks and sensor design according to the protocol proposed by [72].

**Figure 15:**
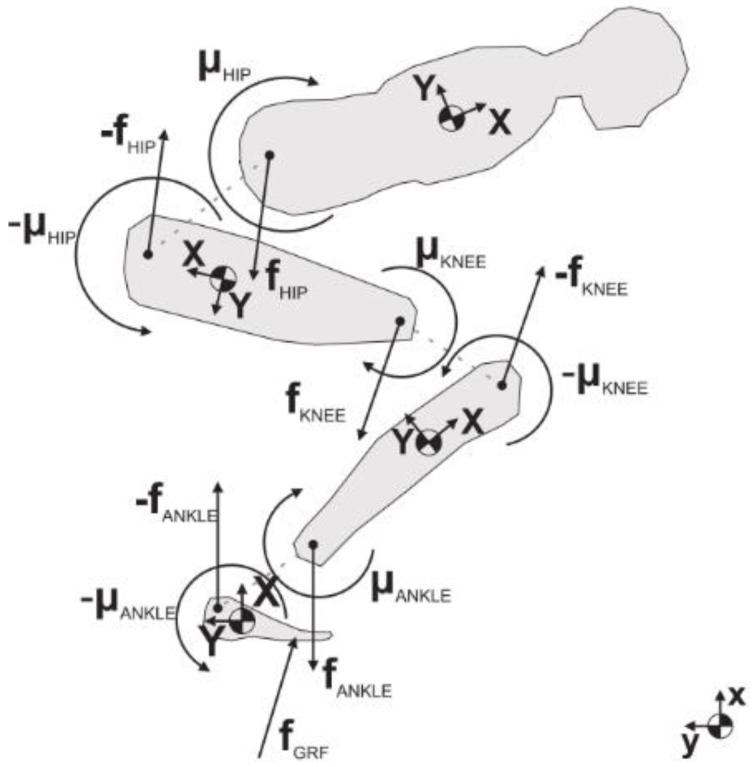
Free body diagram for each segment according to the model proposed by [72].

A posture similar to a squat is the one assumed when performing Ski Jumping. A detailed analysis of GRF and joint forces when performing this sport was conducted by Logar and Munih [72]. They developed a procedure to estimate GRF and joint forces by exploiting kinematics information from body-worn IMUs. The tests were performed in a lab environment with a force platform installed in proximity to the jump area that allowed for the direct measurement of GRF during the take-off (pushing) phase. Ten IMUs were placed on the body of the subject as shown in Figure 14. The IMUs were composed of a tri-axial accelerometer and tri-axial gyroscope with range ±8 g, sampling frequency 400 Hz. Data was logged on-board on each IMU and synced by means of a wireless signal. The human body was modelled as a link segment model composed of four segments as shown in Figure 15. Each segment was assumed as a rigid body with constant mechanical properties. A subject calibration procedure was needed to make the measurements independent of IMU fixation point and position. The procedure involved: (i) Measuring the initial orientation of each segment; (ii) Measuring and integrating the angular velocity of the segment during motion; (iii) summing initial orientation and integrated angular velocity. The initial orientation was estimated during the in-run phase where all the body segments were assumed to have comparable accelerations, the subject kept a squat-like posture and the orientation of feet/skis was assumed as the reference orientation for all the other segments of the model. The anthropometric parameters (mass, size, moment of inertia) were obtained from statistical tables. The inverse dynamics of the proposed model can be solved according to two approaches: bottom-up and top-down. In both cases it is supposed that the only external forces acting on the body are the GRF. In the bottom-up, GRF measured by the force plate are needed. By taking advantage of external GRF, the forces and moments on each body segment can be computed according to Newton-Euler equilibrium equations. In the top-down, the GRF are unknown. The internal forces acting on the upper part of the body are estimated from the acceleration and Newton’s equation. Then the internal forces on the other body segments are progressively computed according to Newton-Euler equilibrium equations. At the foot, the GRF is the quantity needed to balance the last equation. In top-down approach, once the GRF is known, the equation can be re-applied in bottom-up direction in order to compute the internal moments acting on the joints.

**Figure 16:**
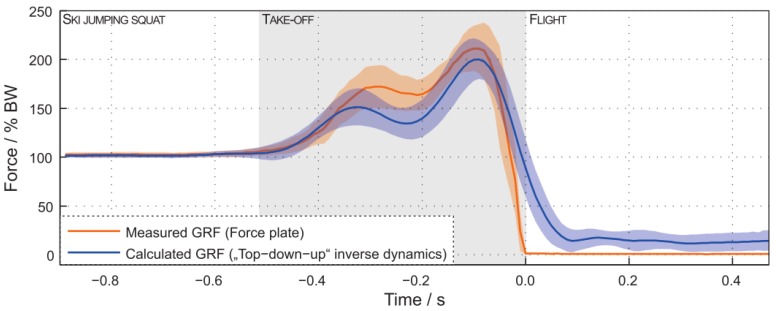
Comparison of the vertical GRF calculated by the IMUs and the one directly measured by the force platform [72].

The validation of this method showed an average RMS error between the estimated GRF and the ones measured by the force plate of 62.5 ± 259 N corresponding to a 9.7 ± 14% deviation [72]. A comparison of the calculated and measured GRF profiles is shown in Figure 16. A portion of the observed differences was attributed to the dimension of the skis with respect to the force platform. The FP reading does not provide the entire GRF but the reading depends on the portion of the skis that is effectively in contact with the FP [72].

The conclusion of the study was that a good similarity between measured and calculated GRF was observed. Thus, the proposed IMU protocol may be considered a promising and easy to use tool for estimating GRF in ski jumping.

The clinical need to measure internal forces and moments acting on the spine and joints, as well as the GRF, during trunk bending led to the protocol proposed by Faber et al. [73]. This protocol was meant to be used in ambulatory environments, relying only on wearable sensors and removing the need of a motion analysis laboratory. The authors used a X-Sens^®^ IMUs in combination with the built-in X-Sens^®^ full body segment model [74,75]. The model used 17 IMUs composed of tri-axial accelerometers, gyroscopes and magnetometers, remotely controlled and triggered. Sampling frequency was 120 Hz. The full body configuration allowed the estimation of the three dimensional GRF acting on the feet segments. The protocol needed a calibration obtained by recording an upright posture and then the acceleration of each body segment during the bending exercise was measured. The moments and the GRF were estimated by means of a top-down approach, using Newton’s second law. The results were compared to the GRF simultaneously measured by a FP and to the internal forces/moments computed taking advantage of the information from the FP and an OS. As observed in previous studies [72], a good agreement between the FP and the IMUs was observed for the profile of vertical component of GRF with a RMS error below 20 N corresponding to the 2% of maximum vertical force [73]. A good agreement was also observed between the peak values of GRF. Instead, the forces on antero-posterior and medio-lateral directions were overestimated by the IMU method. Regarding the internal moments on L5/C1, the maximum RMS error was below 10 Nm corresponding to the 5% of the peak extension moment [73]. The main inaccuracies observed were attributed to the rigid body assumption and to the mass of each body segment that was assigned according to a statistical model based on percentages of the total body mass [73]. Thus, the masses used for computation at may not well represent the masses of the actual body segments of each subject. In agreement with previous studies, the authors concluded that inertial motion capture is a good candidate for GRF and internal moment estimation in ambulatory settings but its validity is limited to the task analysed, i.e., the trunk bending. Further study is required for the analysis of other tasks.

**Figure 17:**
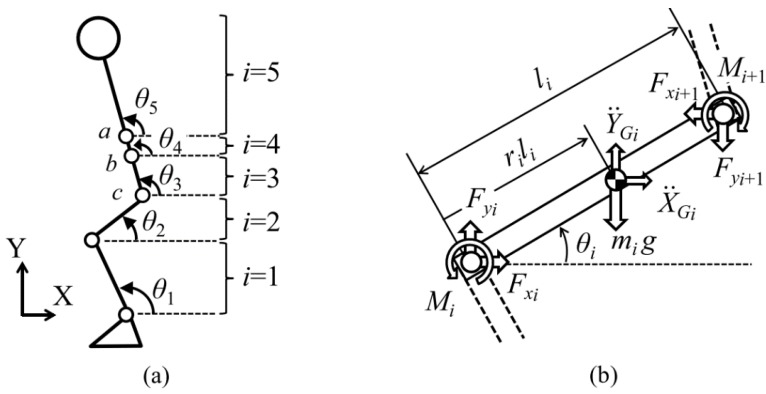
Five-links biomechanical model (**a**) and free body diagram (**b**) of the method proposed by [76]. The inclination angle of each segment is defined on the sagittal plane and the internal forces and moments are represented on the free body diagram.

**Figure 18:**
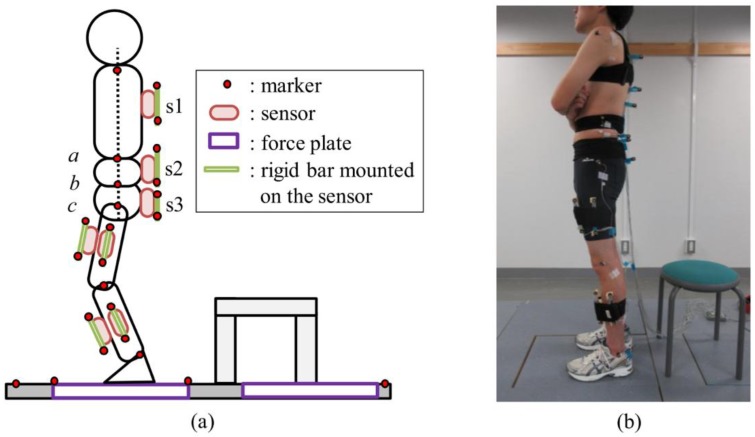
The experimental setup proposed by [76]. (**a**) Representation of body segments and sensors, (**b**) Landmarks and sensors worn by the subject.

The sit to stand and squat tasks were investigated by Kodama and Watanabe [76]. Their goal was to estimate internal joint moment, GRF and CoP relying on IMU kinematic recordings. They tested three body models: a five-links model, a four links model and a three links model. The difference was in the number of segments representing the trunk [76]. The five links model is depicted in Figure 17. The other models were obtained by merging the segments composing the trunk. Inertial parameters of each segment were assigned according to direct measurements on the subjects, statistical distribution methods reported in the literature [77,78] and Human Body Database [79]. The joint moments were estimated by solving the equations of motion of each segment whose free body diagram is depicted in Figure 17 and the GRF were estimated as the sum of products of the acceleration of each segment and the mass of that segment, as in previous methods. The CoP was calculated by using the equation of rotational motion of the foot segment and the forces and moments acting on that segment [76]. Seven inertial sensors were mounted on the trunk, thighs and shanks, as shown in Figure 18. The IMU were custom made wireless sensors with a sampling frequency of 100 Hz. The motion was simultaneously recorded by an OS and two FPs. The subjects then performed the squat and sit-to-stand tasks under investigation. The results showed no significant differences between the 5-link and 4-link models while the worst results were observed for the 3-link model. The reason of the poor results was attributed to the approximation in computing the lever arm for the trunk segment. Therefore, the authors recommended the 4-links model. Regarding the estimation of GRF via the top-down method, no differences were observed between inertial motion capture and OS. Average RMS errors in horizontal, vertical GRF and CoP were respectively 10 N, 15 N and 2 cm. Thus, the method could be useful for practical applications when FPs are not available.

As the model proposed by [76] was based on multiple body segments, it needed an extensive use of standard tables to compute the mechanical parameters of each segment. Such statistical values may introduce inaccuracies in the estimation of the quantities of interest as they may not well represent certain population. This is a common problem for all those methods that require knowledge of inertial properties of body segments.

Setuain and colleagues [80] also evaluated vertical jumping by means of a single IMU placed on the lumbar spine. The aim of their work was to determine the reliability of such method and to its validity in comparison to force plate measurements. The GRF was computed from the acceleration by means of Newton’s law as in previous studies and the measurement of vertical velocity profile allowed the identification of the jump phases. As in previous studies, a good correlation was observed between the estimated vertical GRF and the GRF directly measured by means of a force platform, and a bias between the measured values was also observed. The greater the force magnitude, the greater the disagreement between the IMU and the FP.

### 3.2. Methods Based on Machine Learning

Using machine learning methods was explored as a modern approach for the estimation of GRF. These methods run on the hypothesis that a relationship exists between the acceleration measured somewhere on the human body and the ground reaction forces. In the case of a biomechanical model, this relationship is represented by the model itself. Machine learning methods do not need an a priori knowledge of the model but they build up their model on the go by using training data acquired in previous experiments. Such models based on artificial neural network (ANN) algorithms need to be trained by a large amount of known output data in order to establish a robust relationship between input and output variables. In literature, there are many studies about the estimation of GRF by means of ANN. Most of them are based on kinematic data acquired by OS or other devices than IMUs [81,82,83,84,85]. These are not discussed in the present work as it is focussed on the use of IMUs.

One of the first works involving the use of ANNs is the one by Leporace et al. [86]. They compared two models based on ANN in order to estimate GRF while walking. Both models were based on three-dimensional accelerometer data. Training and control data was provided by a force platform. The healthy subjects were instructed to walk at a self-selected speed while wearing a three-dimensional accelerometer attached to the distal and anterior part of the shank. Range of the accelerometer was ±6 g and the sampling frequency was 1 kHz.

The error analysis suggested that both the models adequately predicted the GRF on vertical, medio-lateral and antero-posterior projections. However, the size of the sample used to train the model is crucial in order to reach accurate results [86].

**Figure 19:**
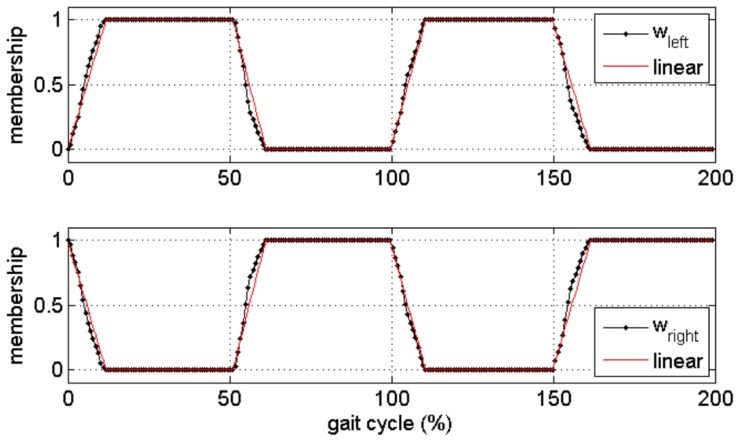
Membership function for the distribution of vertical GRF among the two feet as determined by [87]. The upper line is the left membership function, representing the left single support phases. The bottom line represents the right membership function. The transients represent the double support.

Guo and colleagues [87] adopted a different approach to estimate the vertical component of the GRF by using the acceleration, directly measured by wearable IMUs, and used it as a proxy variable. The aim of the study was to find a relationship between the acceleration, used as a proxy variable, and the forces measured by means of pressure insoles without regard to a biomechanical modelling. The relationship was assumed as non-linear. To achieve this goal, the authors used an orthogonal forward regression algorithm, i.e., a machine learning approach. The vertical GRF measured by pressure insoles were used to train the model that was subsequently used to predict forces from the accelerations. The IMU used were composed of tri-axial accelerometers, gyroscopes and magnetometers, with a sampling frequency of 128 Hz and range ±6 g. Only the acceleration information was used to train the model. In the same study the authors also tested different placements for the measuring IMU on the subjects’ back: L5, C7 and forehead. Data synchronization was obtained by a vertical jump before trials and, since the acquisitions were relatively long, the time series were re-aligned every two minutes [87]. This proposed approach is in contrast with approaches based on detailed biomechanical models and it is claimed to simplify modelling and data acquisition strategies. Furthermore, relying only on acceleration signals makes the model free from disturbances due to gyroscope drifts and magnetic distortion. The identification of stride phases relied on pressure insoles and the exact knowledge of stride events and their sequence helped to determine which foot was in contact with the ground. Thus, the recorded acceleration *a* could be easily decomposed into its left and right components. During double support, the vertical GRF was approximated by linear interpolation and a membership function, *w*, as described in Equations (12) and (13). The left and right components of the acceleration *a* were obtained according to Equations (14) and (15). The final membership function is depicted in Figure 19.

(12) $$w_{left}=\frac{GRF_{left}}{GRF_{left}+GRF_{right}}$$

(13) $$w_{right}=\frac{GRF_{right}}{GRF_{left}+GRF_{right}}$$

(14) $$a_{left}=a\cdot w_{left}$$

(15) $$a_{right}=a\cdot w_{right}$$

**Figure 20:**
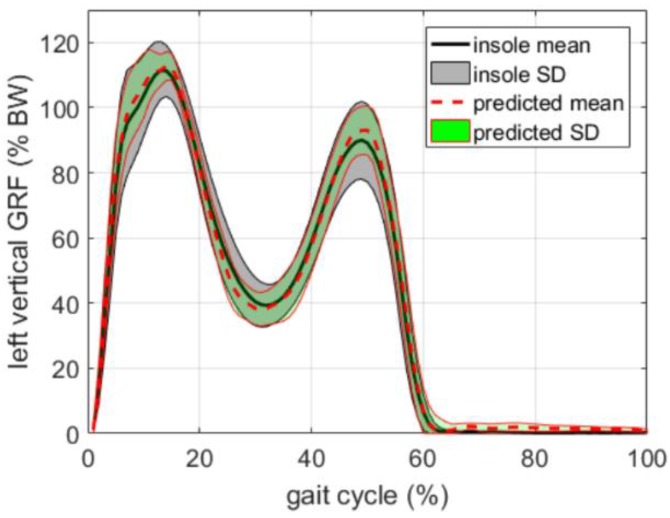
Vertical GRF profile predicted by the method proposed by [87] and the one directly measured by pressure insoles.

The method was tested for walking tasks in outdoor settings. The optimum sensor placement was found to be the L5 which corresponded to the minimum model prediction error and the GRF could be predicted with an accuracy of 3.8%. In the case of cervical (C7) and forehead placements, the prediction error was higher (>4.0%) [87]. In general, the prediction accuracy of the proposed method was comparable to the direct measurement of the vertical GRF by means of pressure insoles, as shown in Figure 20. The results were strongly dependent on the identification of stride phases that was achieved by using pressure insoles. The gait events may also be obtained by an IMU placed at the pelvis level [88], but this approach may increase the inaccuracy in the estimation of GRF [87]. Furthermore, in this study, the analysis was limited to the vertical component of GRF and the method should be tested for the prediction of medio-lateral and antero-posterior components. Other common daily activities should be tested.

**Figure 21:**
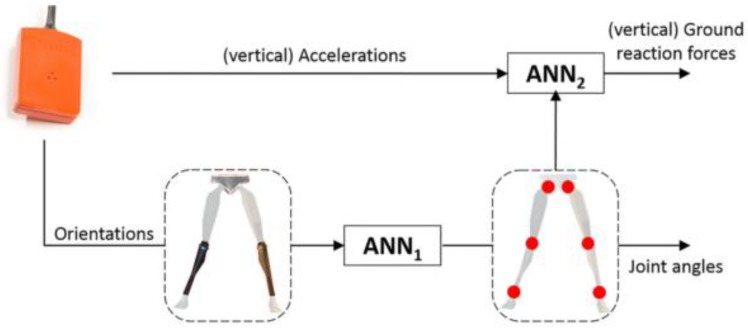
Model based on the two artificial neural networks (ANN) and its training from IMU data. The two ANNs sequentially estimated kinematics and kinetics [89].

A modern work is the one by Wouda et al. [89] that estimated the vertical GRF during running using only three inertial sensors placed on the lower legs and pelvis. The approach was based on two concatenated neural networks opportunely trained. The first one mapped the measured accelerations to lower body joint angles (kinematics). The data were subsequently passed to a second neural network whose aim was to estimate the vertical GRF. This architecture, depicted in Figure 21, allowed for the independent training of the two networks and the selective re-training of each network in case of changes in external conditions. Running test trials were recorded by an instrumented treadmill equipped with a one-dimensional force plate. The GRF measured by the force plate was used for model training and reference. Reference kinematic data was simultaneously recorded by: (i) an OS and (ii) a full body (17 IMUs) inertial motion capture system. The Xsens MVN link model [75] was used to solve the whole body kinematics. Sampling frequency was 240 Hz for the recording and it was resampled to 120 Hz for further processing. The two stages reconstruction was implemented in MATLAB (Mathworks, USA), using the “Neural Network Toolbox” and the outcomes of the model were compared to the complete kinematic and kinetic data obtained by the OS and the instrumented treadmill.

**Figure 22:**
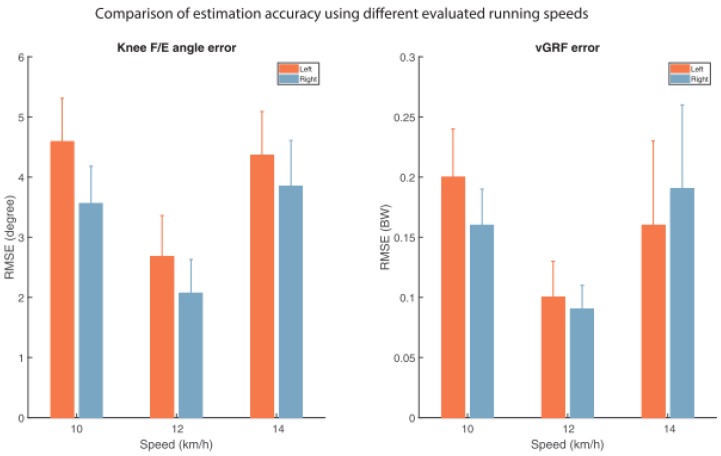
Accuracy of the estimated GRF and knee flexion/extension for different running speeds using single-subject training [89].

When trained with subject-specific data, the model produced excellent results in matching the actual force profiles measured by the treadmill. In this case, the knee flexion/extension angles were estimated with an accuracy <5° while, ground reaction forces were estimated with an accuracy <0.27 BW. The test was repeated at different speeds and the best results were observed at a running speed of 12 Km/h (Figure 22).

**Figure 23:**
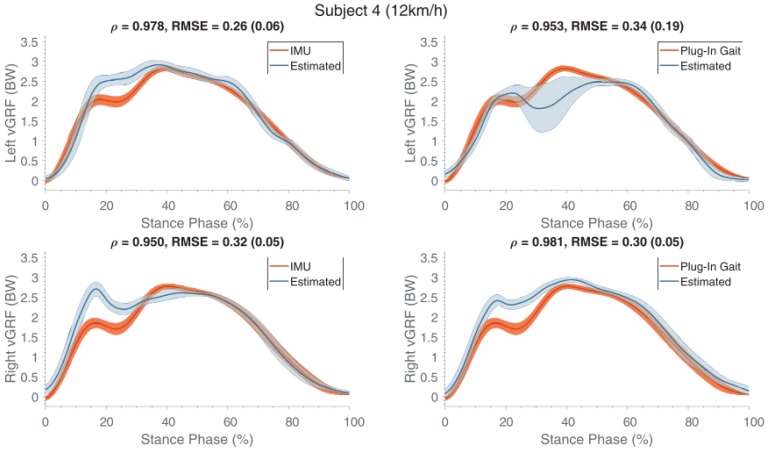
The estimated GRF profiles are compared to the respective reference profiles. Reference profiles were classified according to the respective reference kinematics (IMU and Plug In Gait joint angle output) [89]. These estimates were obtained using training datasets from different subjects. Left forces are depicted on the first row, while right stances are on the bottom row. At the top of each graph it is reported the comparison between the curves in terms of: the Pearson correlation coefficient, the RMSE and its standard deviation [89].

The major issue observed in this approach was the need of a training phase for each subject, as the best results were observed when using training data from the same subject. Training data from other subjects may be used, but in this case a decreased performance of the model has to be expected [89]. Figure 23 shows the force profiles of a subject estimated using training data from the other subjects. Furthermore, the estimation of GRF was limited to its the vertical component, due to the one-component force platform used to collect training data. In general, the estimation of medio-lateral and antero-posterior components of GRF from kinematic data has poor accuracy [47] but the method proposed by [89] may be trained with multi-component data and implemented to estimate lateral components of GRF.

## 4. Summary

The papers reviewed and discussed in the present work are summarized within Table 1. The most important information of each experiment are reported as well as the most important remarks and discoveries highlighted in each paper. Accuracy information, in terms of RMSE, correlation or other measures reported in the original papers were listed as well.

## 5. Conclusions and Final Remarks

Ground reaction forces play an important role in gait analysis and in functional evaluation to study kinetic interaction with the ground. GRF are also necessary to estimate internal joint forces and moments by means of inverse dynamics. The most common and most reliable method to measure GRF is by using force platforms or treadmills instrumented with force sensors. Such instruments require dedicated spaces, such as the motion analysis laboratories, and skilled operators. Thus, portable methods represent an attractive alternative that is worth studying. Non-invasive wearable sensors may be used in ambulatory environments, or on the field for monitoring training and improvements of athletes or performers.

Among the wearable sensors currently available, the most reliable are the ones that allow the direct measurement of GRF. Examples are: the pressure mapping insoles [90,91], wearable load cells [18,92] or ad-hoc designed pressure sensing devices [82]. However, direct measurements have several drawbacks: (i) the sensors are worn under the foot compromising the foot-ground interaction; (ii) sometimes the sensors may change the rigidity of the shoes making them unsuitable for running or jumping tasks; (iii) mechanical and repeated stress of the sensors is high, thus sensors can wear out or break down easily; (iv) sensors may cause discomfort during training. For these reasons, the possibility to indirectly estimate GRF from kinematic data and inertial measurements saw a growing interest in scientific research.

The literature analysis showed that GRFs can be predicted from IMU data by using a biomechanical model in conjunction with Newton’s second law of motion, or a machine learning approach. Vertical GRF can be accurately predicted in case of the single stance, but during double support the forces and moments under each foot cannot be easily determined.

The most critical aspects in estimating GRF from kinematic data were identified in:

(1) The number of sensors/body segments required for the biomechanical modelling
(2) Knowledge of the inertial properties of each body segment
(3) Determining the antero-posterior and medio-lateral components of GRF
(4) Determining the GRF acting on each foot in double support conditions and evaluating loading asymmetry
(5) Even if a correlation between predicted and directly measured GRF exists, it is difficult to estimate the absolute value of peak force.

Increasing the number of sensors means having a better knowledge of motion and acceleration of each body part, leading to a more accurate estimation of GRF. On the other hand, reducing the number of sensors would dramatically simplify subject preparation, data acquisition and subject’s comfort. Approaches based on only one sensor were validated, having the sensor placed either on the sacrum or on the hip but they still have some limitations, mainly due to the poor accuracy. When a multi-segment body model is used, the distribution of inertial quantities is obtained by means of standard tables that assign percentages of the body weight to each segment. This approach may not properly represent each subject and it is a significant source of uncertainty.

According to our literature survey, most of the reviewed papers validated the estimation of the vertical component of GRF that was acceptable in most of the cases, while a few focussed on the lateral components and found a poor reliability in the estimation of such quantities. This result was attributed to the lower absolute values observed for the lateral components of force. Moreover, while the correlation between the body acceleration and the vertical GRF were found to be good in most of the cases, the absolute values of the GRF were not estimated correctly by the IMU method. This may be attributed to inaccuracies in the inertial properties of the models, impact attenuation effects of the shoes and/or sensor placement.

The estimation of GRF in double support is probably the most critical aspect as it is difficult to distribute the forces among the feet. In case of single support, all the estimated force can be attributed to the foot in contact with the ground, which can be identified by means of some techniques based on inertial data. Double support does not occur in tasks such as running where the load is alternatively transferred on each foot. Other tasks such as walking, jumping or training in general have double support phases. Thus, some techniques to assign the estimated load on each foot were proposed, such as a transfer function [44] or a membership function [87]. However these remain statistical models that may not accurately represent each case and their use is restricted to the walking task. When an accurate knowledge of double support forces on each foot is needed, the use of wearable force sensors is recommended.

ANN were proved to be a good flexible tool for nonlinear modelling and versatile for the prediction of GRF. In fact, the use of neural networks simplifies modelling and data acquisition strategies and, since most of the models rely only on accelerometer data, these methods are immune of magnetic disturbance. The drawback of ANN is that they are sensitive to the chosen input parameters, are computationally expensive and require a large amount of data to train the system, in order to reach an acceptable accuracy. ANN are a promising tool for the estimation of the lateral components but this was not validated in the papers examined. Appropriate ANN configuration associated to a multi-segment body modelling may allow to improve the estimation of GRF in double support as well as the loading asymmetry. Further studies should be aimed to increase the accuracy of predicted GRF. The combination of methods based on both biomechanical modelling and machine learning seems a promising way to increase overall accuracy, even in the estimation of lateral components of GRF. Furthermore, hybrid methods based on the concurrent measurement of kinematics and forces by means of miniature wearable sensors should also be explored.

The design of a small non-invasive wearable system or sensor network to estimate GRF represents a significant research challenge. Such a device will enable smart monitoring of training and of injuries or fatigue related to repeated loads on the lower limbs.

## Figures and Tables

**Table 1:** List of the papers discussed.

Reference	Year	Task	No. of Segments	Sensor Type/IMU	Sensor Positioning	Subjects Studied	Method	Reported RMSE or Other Inaccuracy Measures (Worst Case)	Outcomes and Remarks
Ohtaki et al. [26]	2001	Gait	5	1D Acc, 1D Gyro	Distal shank and thigh	Healthy adults	Newton’s Law of motion	Vertical: 0.31 ± 0.012 N/BW Horizontal: 0.076 ± 0.031 N/BW	Gait phase identification. Spectral analysis of acceleration.
Elvin et al. [65]	2007	Vertical jump	2	1D Acc.	Shank	Male athletes	Correlation	Correlation R^2^ = 0.748	Correlation between peak GRF and peak tibial acceleration. Computation of the flying time.
Neugebauer et al. [28]	2012	Walking, running	1	2D Acc.	Iliac crest of the right hip	Healthy teenagers	Statistical Model.	9.0 ± 4.2%	Estimation of peak ground reaction force
Neugebauer et al. [30]	2014	Walking, running	1	3D Acc.	Iliac crest of the right hip	Healthy adults	Statistical model	Vertical: 8.3 ± 3.7% Braking: 17.8 ± 4.0%	Estimation of peak vertical and peak braking ground reaction forces. Acceleration of hip does not estimate correctly GRF. Worst case: running.
Howard et al. [67]	2014	Counter and drop jump	1	3D Acc.	Pelvis	Healthy adults	Newton’s Law of motion	Counter jump: 35.8% Drop jump: 53.6%	Estimated GRF did not match the measured GRF.
Wundersitz et al. [31]	2013	Running, direction change	1	3D Acc.	Upper back, T2	Healthy adults	Newton’s Law of motion	~24%	Acceleration signal needed to be smoothed.
Charry et al. [37]	2013	Running	2	3D Acc.	Medial tibia	Healthy adults	Correlation	8.28%	Implemented gait events identification. Logarithmic correlation observed between acceleration and peak GRF.
Pouliot-Laforte et al. [68]	2014	Vertical jump	1	3D Acc.	Right Hip	Children and teenagers with “osteogenesis imperfect”	Newton’s Law of motion	31%	Good correlation between the GRF estimated and the one directly measured.
Min et al. [71]	2015	Squat	3	3D Acc, 3D Gyro, 3D Mag.	Lumbar spine, thigh, shank	Healthy adults	Inverse dynamics/Newton’s Law of motion	R = 0.93 0.02 BW	High accuracy of estimated GRF. High correlation between acceleration and GRF.
Logar and Munih [72].	2015	Ski Jumping	10	3D Acc, 3D Gyro, 3D Mag.	Total body tracking	Athletes–ski-jumpers	Biomechanical model and inverse dynamics.	12 ± 13%	Required calibration procedure. Good similarity between measured and calculated GRF.
Meyer et al. [39]	2015	Walking, jogging, running, landing and other tasks	1	3D Acc.	Right hip	Healthy Children	Newton’s Law of motion	R = 0.89	Good correlation between acceleration and measured GRF although GRF were overestimated by accelerometer method.
Yang et al. [44]	2015	Walking	7	3D Acc, 3D Gyro	Trunk, thigh, shank, foot.	Healthy adults	Biomechanical model 3D	R = 0.95 66 N	Estimation of the Intersegmental forces and GRF. Identification of walking cycle.
Leporace et al. [86]	2015	Walking	1	3D Acc.	Shank	Healthy adults	Machine learning	Vertical: 5.2 ± 1.7% BW Antero-Posterior: 5.4 ± 1.8% BW Medio-Lateral: 13.0 ± 6.1% BW	Good prediction of all the components of GRF.
Faber et al. [73].	2016	Bending	17	3D Acc, 3D Gyro, 3D Mag.	Full body	Healthy adults	Biomechanical model/Newton’s law.	20 N	Calibration needed. The full body configuration allowed to estimate the three dimensional GRF. Good agreement observed between estimated and measured forces.
Kodama and Watanabe [76]	2016	Sit to stand, squat	7	3D Acc.	Trunk, Pelvis, thigh, shank	Healthy adults	Biomechanical model/Newton’s law.	Vertical: 15 N Horizontal: 10 N	Estimated internal forces/moments, GRF and CoP. Good estimation of GRF. Main limitation due to statistics used to determine inertial properties of body segments.
Setuain et al. [80]	2016	Vertical jump	1	3D Acc, 3D Gyro, 3D Mag.	Lumbar spine	Healthy adults	Newton’s Law of motion	19% R = 0.93	Identification of jump phases from velocity profile. Good correlation between acceleration and force platform, but disagreement between values.
Karatsidis et al. [45]	2017	walking	17	3D Acc, 3D Gyro, 3D Mag.	Full Body	Healthy adults	Biomechanical model	29.6%	Use of smooth transition function to determine GRF in double support.
Gurchiek et al. [56]	2017	Acceleration and change of direction	1	3D Acc, 3D Gyro, 3D Mag.	Sacrum	Healthy adults	Newton’s law.	182.92 N R = 0.53	3D GRF. Static calibration needed. Poor results for the lateral components of force.
Raper et al. [59]	2018	Running	1	3D Acc.	Medial tibia	Professional Athletes	Newton’s law.	16.04%	IMU underestimates the force, but good correlation with the direct measurement.
Aurbach et al. [60]	2017	Gait	15	3D Acc, 3D Gyro, 3D Mag.	Full body	Healthy adults	AnyBody™ musculoskeletal model.	15.60 ± 12.54%	GRF and ankle internal forces.
Guo et al. [87]	2017	Gait	1	3D Acc.	L5, C7, Forehead	Healthy adults	Machine learning.	5.0%	Membership function to identify GRF during double support. Good estimation of GRF. Gait phase identification was dependent on pressure insoles. L5 is the best placement.
Wouda et al. [89]	2018	Running	3	3D Acc, 3D Gyro, 3D Mag.	Pelvis, shank.	Athletes/runners	Multi stage machine learning.	0.27 BW	Minimal sensor setup. Only vertical GRF was estimated. Excellent results when using training data from the same subject.
Thiel et al. [62]	2018	Sprint running	2	3D Acc, 3D Gyro, 3D Mag.	Shank	Athletes/sprinters	Linear modelling. Empirical parameter estimation.	33.32%	Estimation of peak GRF by linear modelling. Method was not reliable for every participant.
Kiernan et al. [63]	2018	Running	1	3D Acc.	Thigh	Athletes/runners	Statistical model/linear regression equation	N.A.	Estimation of peak GRF. Relation between peak GRF and potential injury. Evaluation of the training level. Use of the lateral component of acceleration to determine which foot is in contact with the ground.
